# Real-world costs per life-year of targeted therapy, incidence, lifetime health impact, and medical costs of renal cell carcinoma in Taiwan

**DOI:** 10.3389/fpubh.2026.1808050

**Published:** 2026-04-15

**Authors:** Chin-Heng Lu, Min-Che Tung, Yen-Chuan Ou, Ying-Ming Chiu, Siu-San Tse, Chia-Che Chang

**Affiliations:** 1 Division of Urology, Department of Surgery, Tungs’ Taichung Metroharbor Hospital, Taichung City, Taiwan; 2 Rong Hsing Translational Medicine Research Center, National Chung Hsing University, Taichung City, Taiwan; 3 Doctoral Program in Translational Medicine, National Chung Hsing University, Taichung City, Taiwan; 4 Department of Nursing, Jen-Teh Junior College of Medicine, Nursing and Management, Miaoli, Taiwan; 5 Department of Allergy, Immunology, and Rheumatology, Tungs’ Taichung MetroHarbor Hospital, Taichung City, Taiwan; 6 Department of Post-Baccalaureate Medicine, College of Medicine, National Chung Hsing University, Taichung City, Taiwan; 7 Department of Life Sciences, National Chung Hsing University, Taichung City, Taiwan; 8 Graduate Institute of Biomedical Sciences, Rong Hsing Translational Medicine Research Center, The iEGG and Animal Biotechnology Research Center, National Chung Hsing University, Taichung City, Taiwan; 9 Department of Medical Laboratory Science and Biotechnology, Asia University, Taichung City, Taiwan; 10 Department of Medical Research, China Medical University Hospital, Taichung City, Taiwan; 11 Traditional Herbal Medicine Research Center, Taipei Medical University Hospital, Taipei, Taiwan

**Keywords:** cost per life-year, cumulative incidence, life expectancy, pharmacoeconomics, real-world data, renal cell carcinoma, targeted therapy

## Abstract

**Background:**

The incidence of renal cell carcinoma (RCC) and its associated economic burden have risen significantly in Taiwan. While targeted therapy has become a standard treatment, its real-world effectiveness and cost-effectiveness remain to be fully evaluated.

**Methods:**

This population-based cohort study utilized the Taiwan National Health Insurance Research Database and Cancer Registry to analyze 14,131 RCC cases diagnosed between 1998 and 2016. Key outcomes included life expectancy (LE), loss of LE, and lifetime medical costs.

**Results:**

The cumulative incidence rate of RCC increased from 0.37 to 0.73% in men and from 0.23 to 0.36% in women. Significant LE loss was observed, particularly in patients under 50 years of age (14.38 years in men; 12.89 years in women). In advanced cases, targeted therapy yielded a slightly higher LE (4.43 years) compared to non-targeted therapy (3.63 years); however, the loss of LE was similar between groups.

**Conclusion:**

The real-world relationship between survival outcomes and lifetime medical costs of targeted therapy in Taiwan suggests suboptimal efficiency under current clinical practice. These findings suggest a need to re-evaluate reimbursement strategies by considering pharmacogenomic heterogeneity, implementing genomic profiling for precision medicine, and transitioning toward more effective combination therapy paradigms.

## Introduction

Renal cell carcinoma (RCC) is the most common type of kidney cancer, originating from renal tubular epithelial cells. This prevalent urinary malignancy accounts for approximately 3% of all malignant tumors and ranks among the top 10 most common cancers worldwide (1). The incidence of RCC varies geographically, with rates in Europe and North America higher than those in Asia and South America (2).

The incidence of RCC has risen steadily each year over recent decades (3). RCC can be cured through surgical resection if diagnosed early. Approximately 65% of RCC patients are initially diagnosed with non-metastatic disease, and the 5-year survival rate for stage I RCC is generally greater than 90%. However, survival rates decline for patients diagnosed at later stages (4). The majority of studies estimate the 5- or 10-year survival rates to evaluate the long-term outcomes of RCC. However, potential confounding factors, such as differences in age and sex distributions across study populations, may limit the comparability of reported survival rates (5, 6). These comparisons are further complicated by temporal changes in medical practice and treatment advances, which may lead to improved survival rates in more recent cohorts (3, 4). Beyond these technical challenges, survival probabilities are often abstract and may be difficult for patients and policymakers to interpret, underscoring the need for more intuitive metrics such as life expectancy (LE) and loss of LE (5, 7).

To minimize the potential underestimation of the loss of LE due to RCC (5), we developed a novel method for the calculation of disability-adjusted life-years (8). Specifically, we estimated the LE of patients with a specific disease using accessible nationwide long-term follow-up data. Differences in LE between individuals diagnosed with disease and age- and sex-matched referents based on national life tables are referred to as the loss of LE or expected years of life lost (YLL) (5, 6). Existing links between national LE databases and health insurance reimbursement databases provide long-term data regarding health care expenditures (6, 9, 10). The development of these statistical methods involving big data linkages has provided a more comprehensive estimation of health policy-related issues (11).

RCC incidence has increased in recent decades, accompanied by a substantial increase in economic burden due to advancements in medical technologies, including targeted therapy and surgical techniques (4, 12–14). However, the majority of studies focus on lifetime costs associated with specific medications, costs per life-year paid by various parties, or annual costs borne by insurance carriers (4, 13, 15). To improve the sustainability of a universal health care insurance system, this study aimed to estimate national trends in RCC incidence rates, lifetime medical costs associated with RCC diagnosis and targeted therapy reimbursed by the National Health Insurance (NHI) program, and loss of LE due to RCC in Taiwan based on real-world data.

## Methods

### Study approval

This study was approved by the research ethics committee at China Medical University Hospital (IRB number: CMUH108-REC2-119). An exemption from informed consent was granted by the IRB because all data provided to the researchers were de-identified and released for research purposes by the Taiwan Cancer Registry, the reimbursement database of the NHI program, and the Taiwan Mortality Registry. All methods were conducted in accordance with the Declaration of Helsinki. All methods were performed in accordance with relevant guidelines and regulations.

### Study population and datasets

The flow diagram of the study cohort is presented in Supplementary Figure 1. The study collected, cross-referenced, and mutually validated the following three datasets: the Taiwan Cancer Registry, from 1998 to 2016, which included all newly diagnosed RCC cases; the reimbursement database of the NHI program; and the Taiwan Mortality Registry, from 1998 to 2017, which was used to extract costs and survival data. These datasets contain data regarding the date of diagnosis, cancer site, histology, survival status, and inpatient and outpatient files.

The cancer site of interest was the kidney (ICD-9-CM code:189.0; ICD-10-CM code: C64). To compare the cumulative incidence rates (CIRs) of common urological cancers, two cancer sites were included: renal pelvis (ICD-9-CM code:189.1; ICD-10-CM code: C65) and ureter (ICD-9-CM code:189.2; ICD-10-CM code: C66).

To explore the benefit of targeted therapy, we performed a sub-analysis of patients with advanced-stage RCC who received targeted therapy, radiation therapy, or chemotherapy. Advanced-stage RCC and those with other catastrophic illnesses were excluded. Targeted therapy, radiation therapy, or chemotherapy data were obtained from the reimbursement database of the NHI program. Targeted therapy included sorafenib, sunitinib, everolimus, temsirolimus, pazopanib, and axitinib. Radiation therapy was identified using NHI reimbursement codes (36001–36,023, 37,001–37,048). Chemotherapy agents included gemcitabine, cisplatin, carboplatin, vinblastine, paclitaxel, docetaxel, and 5-fluorouracil.

### Cumulative incidence rate

The CIR was defined as the probability that an individual will develop a specific disease within a specified time frame. The CIR was calculated for individuals aged between 20 and 79 years (CIR_20–79_) to estimate the lifetime risk associated with RCC. Over a period of 2 or 3 consecutive calendar years, we calculated age- and sex-specific incidence rates using the number of new cases as the numerator and the corresponding mid-year population at risk, based on census data from the Ministry of the Interior in Taiwan, as the denominator.

The overall CIR_20–79_ value can be calculated as 1 minus the exponential of the cumulative product of age- and sex-specific incidence rates, as follows:

$${\mathrm{CIR}}_{20-79}=1-\exp(-\sum_{i}(IR_{i})(\Delta t_{i}))$$

Where CIR_20–79_ is the sex-specific cumulative incidence rate for the population age range of 20–79 years; i = 20–49, 50–59, 60–69, 70–79; IR_i_ is the incidence rate of the i-th age group; and Δt_i_ is the range of the i-th age group.

### Estimation of life expectancy and loss of life expectancy

The survival curves for RCC, stratified by sex and age, were estimated using the Kaplan–Meier (KM) test, and follow-up was recorded until the patient was either deceased or censored on 31 December 2017. To estimate LE after receiving an RCC diagnosis, we applied the free R package iSQoL2 to extrapolate the lifetime survival curve. iSQoL2 uses a semiparametric survival extrapolation method that was proposed and verified by Hwang et al. (16), and was subsequently applied to the estimation of survival following liver cancer by Kuo et al. (17), stroke by Cheon et al. (18), and rheumatoid arthritis by Chiu et al. (19). We briefly outline these methods below.

The extrapolation method can be divided into three phases. First, we created an age-, sex-, and calendar year-matched reference population based on the life tables obtained from Taiwan National Vital Statistics using Monte Carlo methods. The lifetime survival functions of these referents were also estimated using the KM method. Second, we calculated the survival ratio between the index and reference populations at each time point, t, and applied a logit transformation. By fitting a restricted cubic spline model, we extrapolated the survival curve of the index cohort for the prediction of 1 month. We omitted the survival data from the first month and used the survival rate of the newly evolved month as the actual 1-month survival, which was used to extrapolate the next month in a rolling-over algorithm, refitting the restricted cubic spline model for each successive month. These processes were repeated until the survival rate of the index cohort fell below 0.01.

LE was calculated as the area under the survival curve of each cohort. The difference in LE values between the index and reference cohorts was considered the loss of LE associated with RCC occurrence.

### Estimation of lifetime health care expenditures for patients

Methods for estimating lifetime medical costs were also proposed by Hwang et al. (16). We collected reimbursement data from the NHI database, including inpatient and outpatient files, summed the monthly costs for all RCC cases, and divided these values by the total number of surviving cases each month to estimate the monthly average health care expenditures incurred following an RCC diagnosis.

We also explored data for deceased cases to identify the month in which health care costs began to rise near the end of life. The mean cost function was estimated as a weighted average of the mean expenditures incurred by patients during the months before their deaths for all the extrapolated survival months. The lifetime health care cost was obtained by multiplying the mean cost function and the survival function and summing the results over the patient’s lifetime.

In this calculation, we also adjusted annual NHI expenditures using the Consumer Price Index. For consistency, the costs of extrapolated months were adjusted using an annual discount rate of 3%. All monetary values are expressed in 2017 dollars (USD) (1 USD = 30.44 TWD). The estimates of lifetime costs were also calculated using the iSQoL2 package.

Given that health-related quality of life data were not available, the economic outcomes in this study are expressed as cost per unadjusted life-year rather than cost per quality-adjusted life-year (QALY).

## Results

A total of 14,131 newly diagnosed cases of RCC were identified for individuals aged 20 to 79 years between 1998 and 2016. The number of cases reported in men was approximately twice that reported in women.

### Cumulative incidence rate

**Figure 1:**
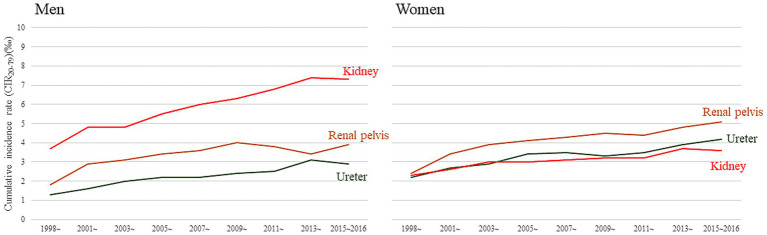
Cumulative incidence rates CIR_20–79_ of three urological cancer types in Taiwan, stratified by location, sex, and calendar year of diagnosis.

Figure 1 and Supplementary Table 1 summarize the dynamic changes in CIR_20–79_ for three common urological cancers stratified by sex. Regardless of cancer type, a consistently increasing trend was observed from 1999 to 2016. Notably, in men, the lifetime risk of RCC was greater than that in women. By contrast, the lifetime risks of renal pelvic cancer and ureter cancer were lower in men compared to women.

**Figure 2:**
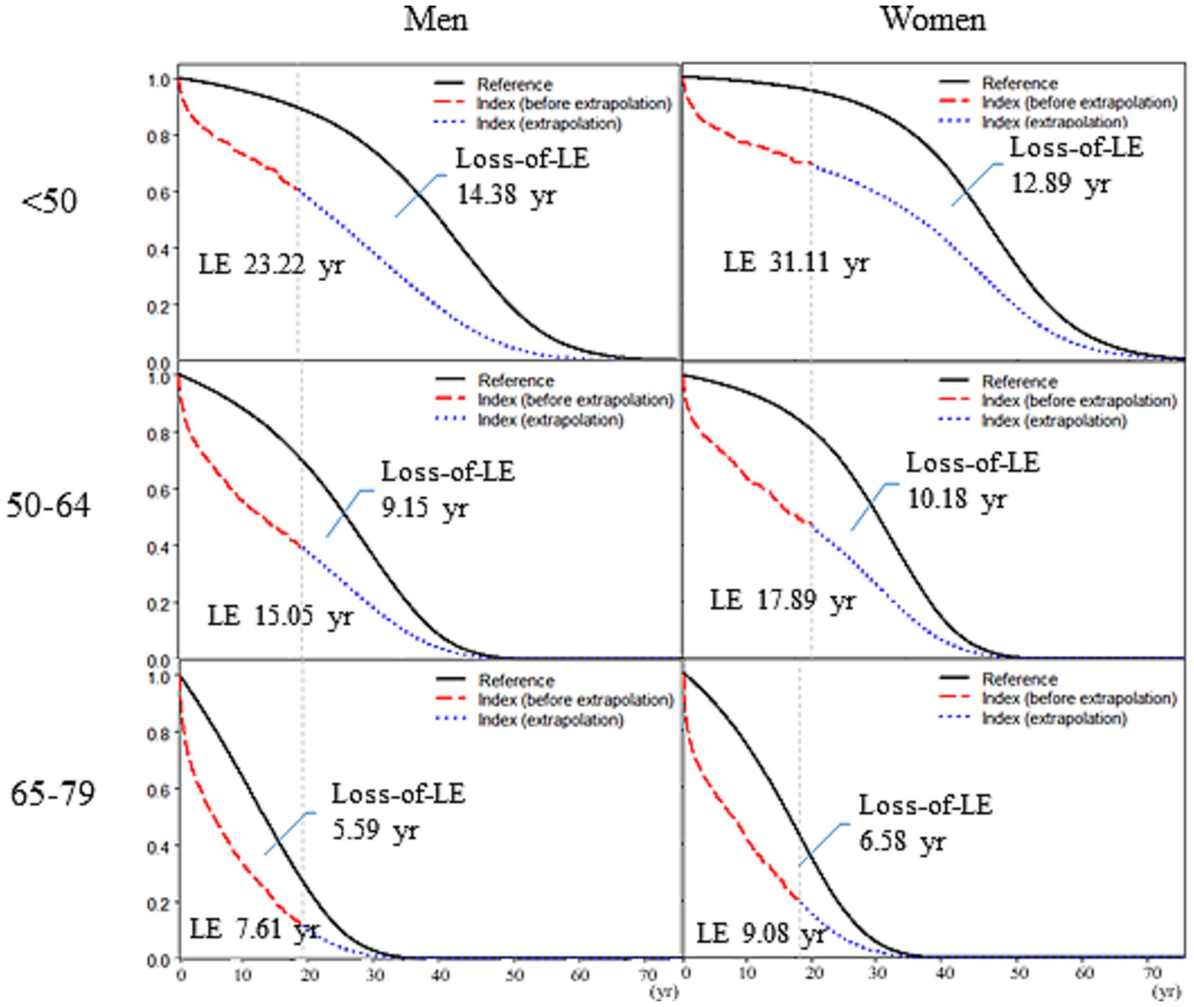
Life expectancy (LE) and loss of LE after diagnosis of renal cell carcinoma (RCC), stratified by sex and age. The black lines represent the survival curves for sex-, age-, and calendar year–matched referents, simulated from Taiwan vital statistics. The reddish dashed lines indicate the actual survival curves among patients with RCC (the index cohorts), whereas the blue dotted lines represent the extrapolated portions.

The incidence rates of RCC, stratified by sex and age, are presented in Figure 2, revealing an upward trend from 1999 to 2016. The incidence rate increased with age, with the highest incidence rate observed in the ≥70-year age group.

### Estimation of life expectancy and loss of life expectancy

Table 1 summarizes the estimated LE and loss of LE stratified by sex and age for RCC. In addition, Figure 2 shows the correlation between LE and loss of LE for all RCC diagnoses. The highest loss of LE was observed among men and women younger than 50 years, who experienced losses of 14.38 and 12.89 years in LE, respectively.

**Table 1:** Life expectancy (LE), loss of LE, lifetime costs, and costs per life-year associated with renal cell carcinoma (RCC) in Taiwan, stratified by sex and age.

Age	N	Deaths, No (%)	LE (SEM)** (in years)	Loss of LE (SEM)** (in years)	Lifetime cost (USD) (SEM)**†	Cost (USD) per life-year†
Men						
<50	2,165	529 (24.4)	23.22 (1.89)	14.38 (1.92)	95,565 (6864)	4,544
50–64	3,716	1,398 (37.6)	15.05 (0.53)	9.15 (0.53)	79,495 (3572)	5,520
65–79	3,499	2,162 (61.8)	7.61 (0.19)	5.59 (0.20)	46,660 (1123)	6,162
Women						
<50	1,101	238 (21.6)	31.11 (2.15)	12.89 (2.13)	93,383 (7889)	3,568
50–64	1,749	567 (32.4)	17.89 (0.81)	10.18 (0.81)	80,838 (5156)	4,770
65–79	1,901	1,045 (55.0)	9.08 (0.3)	6.58 (0.32)	48,264 (2207)	5,358

*Surgical treatment only: during the first year after diagnosis.

**SEM, standard error of the mean.

†1 USD = 30.44 TWD (2017).

Cost per life-year is a point estimate calculated by dividing the mean lifetime cost by the mean life expectancy (discounted at 3%). The variability of the primary parameters is represented by the SEMs provided in the respective columns.

### Estimation of lifetime health care expenditures for patients

Patients diagnosed with RCC at a younger age generally incurred higher lifetime costs, regardless of sex, while those diagnosed at older ages incurred higher average annual treatment costs (Table 1), with values of approximately USD 94,000, 80,000, and 47,000 for the age groups <50, 50–64, and 65–79 years, respectively. Within the same age groups, the average annual costs appeared higher for men than for women.

The proportions of patients who ever received targeted therapy were higher in male patients over 50 years old, particularly among those aged 50–64 years across all RCC cases (Table 2). Although monthly healthcare expenditures generally increased with age, the trend appeared to reverse in advanced-stage RCC patients. The cost per month was higher in the targeted therapy group (Table 2).

**Table 2:** Comparison of monthly medical costs* (cost/month) during the follow-up period for advanced-stage renal cell carcinoma patients under different treatments, stratified by sex, age, and targeted therapy in Taiwan.

Age (years)	No. of (%) patients with targeted therapy	Monthly cost after targeted therapy (USD)*	No. (%) of patients without targeted therapy	Monthly cost after chemotherapy or radiation therapy (USD)*
Male				
<50	251 (11.6)	3,479	272 (12.6)	2,737
50–64	670 (18.0)	3,248	609 (16.4)	2,367
65–79	495 (14.1)	2,934	724 (20.7)	2,158
Female				
<50	106 (9.6)	3,153	160 (14.5)	2,005
50–64	192 (11.0)	2,997	291 (16.6)	2,031
65–79	185 (9.7)	2,607	332 (17.5)	2,033

Patients without targeted therapy received chemotherapy or radiation therapy instead.

*1 USD = 30.44 TWD (2017).

Table 3 shows that LE in RCC patients was higher in the targeted therapy group (4.43 years) compared to patients who received chemotherapy or radiation therapy without targeted therapy (3.63 years). The loss of LE was similar between targeted therapy and non-targeted therapy groups, but patients receiving targeted therapy incurred approximately double the cost per life-year (USD 16,702 vs. 8,773). The proportions of deaths due to cancer was 93.63% in the targeted therapy group and 94.1% in the non-targeted therapy group.

**Table 3:** Life expectancy (LE), loss of LE, lifetime cost, and cost per life-year of advanced-stage renal cell carcinoma, stratified by targeted therapy.

Targeted therapy	N	Deaths, No (%)	Men, No (%)	Age at diagnosis, mean (SD)	LE (SEM)* (in years)	Loss of LE (SEM)* (in years)	Lifetime cost (USD) (SEM)^*,†^	Cost (USD) per life-year†
Yes	807	581 (72.0)	604 (74.9)	59.59 (11.41)	4.43 (0.54)	19.34 (0.61)	73,752 (6,455)	16,702
No**	396	322 (81.3)	278 (70.2)	60.48 (12.94)	3.63 (0.72)	19.41 (0.84)	31,501 (2,835)	8,773

Advanced-stage RCC and those with other catastrophic illnesses were excluded.

*SEM, standard error of mean.

†1 USD = 30.44 TWD (2017).

**Patients received chemotherapy or radiation therapy instead of targeted therapy.

Cost per life-year is a point estimate calculated by dividing the mean lifetime cost by the mean life expectancy (discounted at 3%). The variability of the primary parameters is represented by the SEMs provided in the respective columns.

## Discussion

In this study, we observed a consistently increasing trend in cumulative incidence rates of RCC in both sexes (Figure 1), which was associated with substantial loss of LE and considerable lifetime financial burden over the past two decades (Table 1). The accuracy of our estimations can be supported as follows:

First, because all patients diagnosed with cancer have been eligible for a copayment waiver from the NHI since 1995, every cancer diagnosis must be validated by at least two specialists to prevent any misuse of this benefit (20). To qualify for a copayment waiver, the cancer diagnosis must be substantiated by pathological proof, ensuring the accuracy of estimated incidence rates.

Second, because the index cohorts have been followed for up to 19 years, which is longer than the typical LE of patients diagnosed with RCC who are older than 50 years of age, relatively few years are required for extrapolation (Table 1 and Figure 2). Our extrapolation method is performed by repeatedly constructing spline models based on the logit of the survival ratio between the index cohort and age-, sex-, and calendar year-matched referents simulated from general population mortality rates, which were corroborated by recent statistical reviews (7, 21, 22). Third, because all patients diagnosed with RCC were waived from copayment, our estimation of lifetime costs reimbursed by NHI is comprehensive.

Fourth, although the effectiveness of healthcare services is directly compared using survival rates in randomized controlled trials, the adoption of real-world data must adjust for differences in age, sex, and medical technology in the calendar year of diagnosis. The comparison of loss of LE, or difference-in-differences, would be less confounded by these issues than LE. Thus, we tentatively conclude that our estimations are relatively accurate and would be useful in the consideration and comparison of cost per life-year across different technologies and diseases.

Although the majority of studies related to long-term outcomes in RCC typically quantify the 5- or 10-year survival rates, these numbers may not be easily comprehensible to laypeople. In this study, we adopted a novel method for estimating LE and the loss of LE to compare across various RCC subcohorts. Table 1 shows that women with RCC generally presented with longer LE than men, particularly those diagnosed in the younger age group, which appears to corroborate previous reports (23–25). However, the loss of LE was similar between men and women, suggesting that the health impacts of RCC are similar between sexes after adjusting for age, sex, and differences in available medical technology at the time of diagnosis.

The majority of previous studies have generally reported cost per year or lifetime costs associated with specific medications used to treat metastatic RCC and have been based on Markov models with underlying assumptions, supplemented by sensitivity analyses for potential health policy decisions (4, 13). In contrast, our team has taken advantage of 19 years of follow-up data (26) and developed a novel method for extrapolating the survival function over the patient’s lifetime. We have successfully estimated lifetime medical costs based on real-world data (16, 27), which could be considered a validation of Markov model predictions.

We found that the lifetime costs of RCC are similar between men and women, but women diagnosed with RCC tend to incur a lower average cost per life-year than men after stratification by age (Table 1). As the patient’s age increased, the costs of caring for RCC also tended to increase (14), which is corroborated by the present study.

Our dataset has included information on the presence of bilateral disease since 2007, which showed only 0.27% (28/10,289 new cases) with both sides affected, a proportion that is much lower than what was reported by Berczi et al. (28). Such a small proportion is unlikely to bias the estimation of overall survival rates or the associated costs.

Table 1 indicates that more than half of our patients were older than 65 years or of working age, as previously reported (29). This finding implies the importance of prevention and early detection of RCC from a young age. In addition to early diagnosis among those with a family history of RCC, efforts should be made to promote behavioral changes to avoid major risk factors of RCC, including smoking, obesity, hypertension, exposure to cadmium, chronic kidney disease, and long-term dialysis (29, 30).

Targeted therapy was first reimbursed by the NHI of Taiwan between 2009 and 2012, which included sorafenib, sunitinib, everolimus, temsirolimus, and pazopanib, followed by the addition of axitinib in 2017, with frequencies summarized in Supplementary Table 2. Among these, sunitinib accounted for 47% of the usage. As the NHI did not reimburse immunotherapy before 2019, all costs incurred in this study do not include such treatments.

The reimbursed targeted therapy was used in metastatic RCC patients. Before the targeted therapy era, these patients received chemotherapy or radiation therapy if the patient refused conservative treatment. This suggests that the stage of RCC was similar between patients who received targeted therapy and those who received chemotherapy or radiation therapy.

Table 2 shows that monthly healthcare expenditures were higher in younger advanced-stage RCC, which may indicate greater treatment tolerance and longer treatment duration in younger patients (Table 2). Table 3 shows that despite a similar loss of LE between treatment groups, patients receiving targeted therapy incurred nearly double the lifetime cost and cost per life-year.

Given these findings, the underlying mechanisms cannot be directly evaluated in our dataset. Several factors may contribute to the results, including differences in patient characteristics, treatment allocation, and potential pharmacogenomic heterogeneity; however, these should be interpreted as hypotheses requiring further investigation.

Looking forward, a more refined assessment of the real-world economic outcomes of targeted therapy in Taiwan should (i) construct more comparable clinical subgroups using more granular tumor stage information (beyond a broad ‘advanced-stage’ definition), (ii) explicitly account for treatment sequencing, as initiation of targeted therapy after prior treatments may be associated with different prognosis and accumulated costs, and (iii) incorporate quality-adjusted outcomes (cost per QALY) and a broader cost perspective when such data become available.

## Limitations

This study has the following limitations that must be acknowledged.

First, due to the unavailability of staging data, detailed treatment information, and clinical indicators such as ECOG performance status were not available from Taiwan’s NHI database, LE, loss of LE, and other parameters could not be further stratified according to differences in disease stage or treatment methods. This limitation introduces the potential for “confounding by indication,” as patients selected for high-cost targeted therapies may possess systematically different baseline characteristics, such as better performance status or fewer comorbidities, compared to those receiving conventional treatments. Early diagnosis and treatment could reduce LE loss, and further studies are warranted to explore potential screening methods to decrease the burdens associated with RCC.

Second, the lifetime costs associated with an RCC diagnosis in our study were calculated according to reimbursement data obtained from Taiwan’s NHI database, which does not include patients’ out-of-pocket expenses. As we have included all reimbursement data after diagnosis, our estimation of lifetime costs is also likely to include reimbursement costs related to other comorbidities and may therefore overestimate the total costs that can be directly attributed to RCC, especially among patients with longer survival rates. Moreover, further studies are warranted to explore the cost-effectiveness of different types of targeted therapy.

Third, because this study did not consider patients’ quality of life, we were unable to estimate the QALY or cost per QALY. Consequently, our primary economic metric should be interpreted as the cost per unadjusted life-year. Future studies must consider both quality of life and other societal impacts associated with the increasing burden of RCC, such as productivity loss and demand for long-term care (11). In particular, future research should evaluate cost per QALY for different targeted therapies. Future studies are warranted to incorporate this information to provide a more comprehensive estimation of the cost-effectiveness of RCC treatment.

## Conclusion

The incidence rates and economic burdens associated with RCC have increased significantly over the past two decades in Taiwan. Our real-world findings highlight the need for health authorities to re-evaluate reimbursement strategies for targeted therapies. Specifically, future policies should consider potential pharmacogenomic heterogeneity, the integration of precision medicine through pharmacogenomic profiling, and the transition from single-agent to more effective combination therapy paradigms to optimize clinical outcomes and healthcare resource allocation.

## Data Availability

The data analyzed in this study is subject to the following licenses/restrictions: the data used in this study were obtained from the Health and Welfare Data Science Center (HWDC), Ministry of Health and Welfare, Taiwan. To ensure participant anonymity, all personally identifiable information was encrypted prior to analysis. Due to legal and ethical restrictions under Taiwanese regulations, these data are not publicly available. Researchers interested in accessing the data must submit a formal application, including a detailed research proposal, to the HWDC through their official website (https://www.apre.mohw.gov.tw/). Access will be granted only upon approval by the HWDC and completion of a data use agreement. Requests to access these datasets should be directed to Health and Welfare Data Science Center (HWDC), Ministry of Health and Welfare, Taiwan, https://www.apre.mohw.gov.tw/.
