# Supplementary material for: Real-world costs per life-year of targeted therapy, incidence, lifetime health impact, and medical costs of renal cell carcinoma in Taiwan
Source: Front Public Health. 2026 Apr 15;14:1808050. doi: 10.3389/fpubh.2026.1808050 (PMC13125099; doi:10.3389/fpubh.2026.1808050)

**Supplementary Materials**

**Supplementary Table 1.** Cumulative incidence rate (CIR)_20–79_ of urological cancer, stratified by location, sex, and year of diagnosis.

| Cancer location |  | **Men** | | | | | | | | |  | **Women** | | | | | | | | |
| --- | --- | --- | --- | --- | --- | --- | --- | --- | --- | --- | --- | --- | --- | --- | --- | --- | --- | --- | --- | --- |
|  | n | 1998– | 2001– | 2003– | 2005– | 2007– | 2009– | 2011– | 2013– | 2015–2016 | n | 1998– | 2001– | 2003– | 2005– | 2007– | 2009– | 2011– | 2013– | 2015–2016 |
| Kidney | 10313 | 3.7 | 4.8 | 4.8 | 5.5 | 6 | 6.3 | 6.8 | 7.4 | 7.3 | 5412 | 2.3 | 2.6 | 3 | 3 | 3.1 | 3.2 | 3.2 | 3.7 | 3.6 |
| Renal pelvis | 5005 | 1.8 | 2.9 | 3.1 | 3.4 | 3.6 | 4 | 3.8 | 3.4 | 3.9 | 5988 | 2.4 | 3.4 | 3.9 | 4.1 | 4.3 | 4.5 | 4.4 | 4.8 | 5.1 |
| Ureter | 3246 | 1.3 | 1.6 | 2 | 2.2 | 2.2 | 2.4 | 2.5 | 3.1 | 2.9 | 4775 | 2.2 | 2.7 | 2.9 | 3.4 | 3.5 | 3.3 | 3.5 | 3.9 | 4.2 |
| Type of renal cell carcinoma | | | | | | | | | | | | | | | | | | | | |
| Sarcomatoid | 140 | 0.004 | 0.057 | 0.084 | 0.089 | 0.064 | 0.125 | 0.082 | 0.048 | 0.121 | 75 | - | 0.021 | 0.046 | 0.043 | 0.063 | 0.031 | 0.053 | 0.029 | 0.063 |
| Papillary | 317 | - | - | - | - | 0.003 | 0.018 | 0.197 | 0.525 | 0.548 | 141 | - | - | - | - | - | - | 0.084 | 0.226 | 0.204 |
| Chromophobe | 430 | 0.001 | 0.043 | 0.117 | 0.178 | 0.214 | 0.241 | 0.287 | 0.332 | 0.359 | 401 | 0.004 | 0.029 | 0.106 | 0.136 | 0.134 | 0.237 | 0.217 | 0.363 | 0.305 |
| Clear cell | 3988 | 0.055 | 0.058 | 0.216 | 0.801 | 1.875 | 2.727 | 3.476 | 3.82 | 4.04 | 1679 | 0.032 | 0.008 | 0.071 | 0.346 | 0.868 | 1.191 | 1.463 | 1.489 | 1.653 |

* Values are risks of lifetime occurrence per thousand if a person has not died of any other diseases between 20 and 79 years of age.

**Supplementary Table 2.** The frequency of each target therapy reimbursed for renal cell carcinoma (RCC) in Taiwan, stratified by sex and age. The NHI (National Health Insurance) of Taiwan has reimbursed the target therapies of sorafenib, sunitinib, everolimus, temsirolimus, pazopanib, and axitinib since October 1, 2009, January 1, 2010, February 1, 2011, January 1, 2012, August 1, 2012, and January 1, 2017, respectively.

| Age | sorafenib | sunitinib | everolimus | temsirolimus | pazopanib | axitinib |
| --- | --- | --- | --- | --- | --- | --- |
| Men |  |  |  |  |  |  |
| <50 | 45 | 161 | 111 | 31 | 22 | 16 |
| 50–64 | 92 | 498 | 256 | 43 | 84 | 42 |
| 65–79 | 68 | 345 | 134 | 32 | 78 | 14 |
| Women |  |  |  |  |  |  |
| <50 | 27 | 68 | 51 | 9 | 8 | 6 |
| 50–64 | 41 | 131 | 85 | 21 | 31 | 8 |
| 65–79 | 22 | 139 | 44 | 11 | 26 | 4 |
| Total | 295 | 1342 | 681 | 147 | 249 | 90 |

**Supplementary Figure 1.** Flow Diagram of Study Cohort


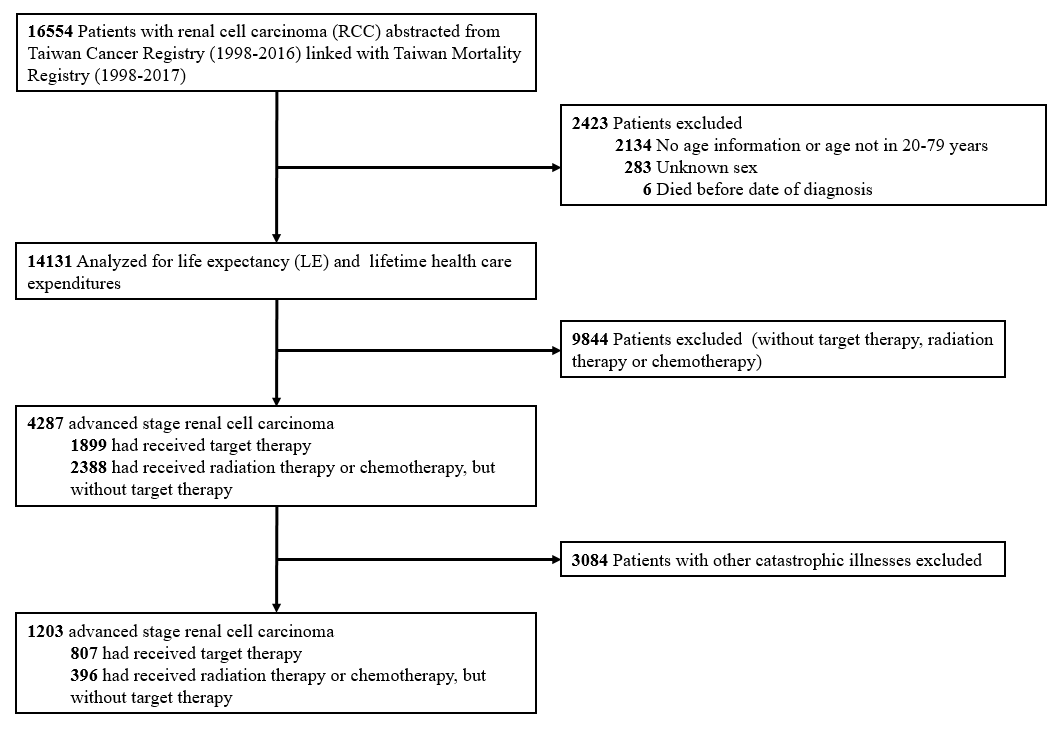


**Supplementary Figure 2** Age-specific incidence rates for renal cell carcinoma (RCC), stratified by sex and calendar year of diagnosis.


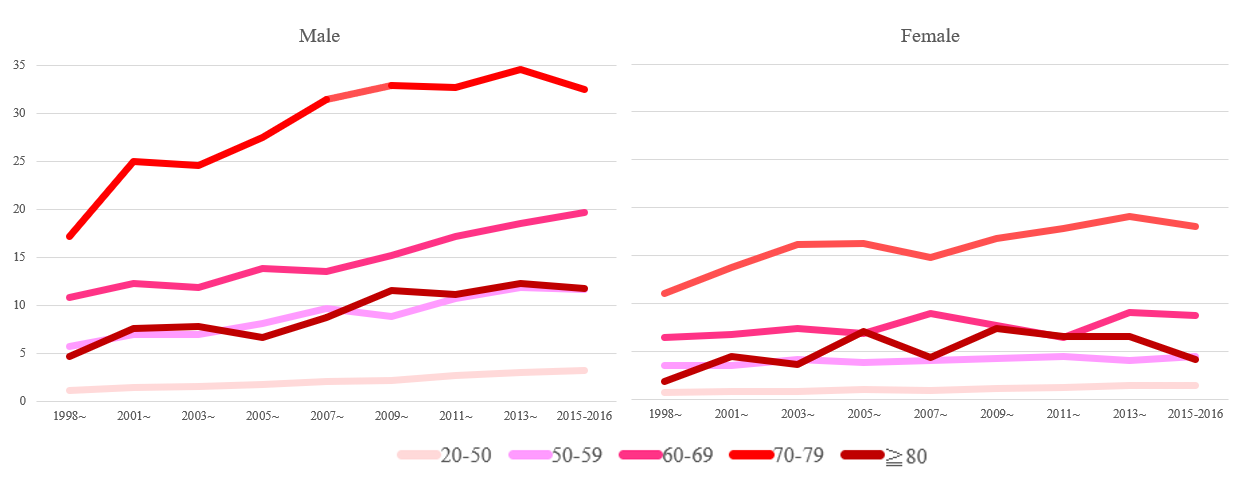

Supplement: Supplementary file 1 [file Data_Sheet_1.DOCX]
